# Supplementary material for: Propofol-based intravenous anesthesia is associated with better survival than desflurane anesthesia in pancreatic cancer surgery
Source: PLoS One. 2020 May 21;15(5):e0233598. doi: 10.1371/journal.pone.0233598 (PMC7241788; doi:10.1371/journal.pone.0233598)
Supplement: S1 Table — (DOC) [file pone.0233598.s001.doc]

1. **“Time since the earliest included patient” was replaced with “Calendar period”**

| **Variables in the Equation** | | | | | | | | |
| --- | --- | --- | --- | --- | --- | --- | --- | --- |
|  | B | SE | Wald | df | Sig. | Exp(B) | 95.0% CI for Exp(B) | |
|  |  |  |  |  |  |  | Lower | Upper |
| Type of anesthesia | -.758 | .246 | 9.494 | 1 | .002 | .469 | .289 | .759 |
| Age | -.021 | .016 | 1.667 | 1 | .197 | .979 | .948 | 1.011 |
| CCI | .517 | .148 | 12.264 | 1 | .000 | 1.677 | 1.256 | 2.240 |
| ASA | .152 | .356 | .182 | 1 | .670 | 1.164 | .579 | 2.337 |
| TNM stage |  |  | 15.056 | 2 | .001 |  |  |  |
| TNM stage II | 1.024 | .393 | 6.806 | 1 | .009 | 2.785 | 1.290 | 6.014 |
| TNM stage III | 1.731 | .456 | 14.383 | 1 | .000 | 5.647 | 2.308 | 13.817 |
| CA19-9 | .806 | .301 | 7.149 | 1 | .008 | 2.239 | 1.240 | 4.043 |
| Methasone use | -.978 | .255 | 14.757 | 1 | .000 | .376 | .228 | .619 |
| Grade of surgical complications _3 group |  |  | 1.881 | 2 | .390 |  |  |  |
| Grade of surgical complications _3 group(1) | .378 | .312 | 1.473 | 1 | .225 | 1.460 | .792 | 2.690 |
| Grade of surgical complications _3 group(2) | .480 | .506 | .902 | 1 | .342 | 1.616 | .600 | 4.354 |
| Chemotherapy | .288 | .251 | 1.312 | 1 | .252 | 1.334 | .815 | 2.183 |
| Metformin use | -1.451 | .494 | 8.634 | 1 | .003 | .234 | .089 | .617 |
| Calendar period 3 group |  |  | 3.768 | 2 | .152 |  |  |  |
| Calendar period 3 group(1) | .547 | .287 | 3.638 | 1 | .056 | 1.728 | .985 | 3.032 |
| Calendar period 3 group(2) | .276 | .378 | .531 | 1 | .466 | 1.318 | .628 | 2.766 |
