# Supplementary material for: Propofol-based intravenous anesthesia is associated with better survival than desflurane anesthesia in pancreatic cancer surgery
Source: PLoS One. 2020 May 21;15(5):e0233598. doi: 10.1371/journal.pone.0233598 (PMC7241788; doi:10.1371/journal.pone.0233598)
Supplement: S2 Table — (DOCX) [file pone.0233598.s002.docx]

| **2. Anesthesiologists * Type of Anesthesia Crosstabulation** | | | | |
| --- | --- | --- | --- | --- |
| Count | | | | |
|  | | Type of anesthesia | | Total |
|  |  | Propofol | desflurane |  |
| Anesthesiologists | 1 | 30 | 0 | 30 |
|  | 2 | 0 | 4 | 4 |
|  | 3 | 21 | 0 | 21 |
|  | 4 | 0 | 7 | 7 |
|  | 5 | 0 | 4 | 4 |
|  | 6 | 1 | 0 | 1 |
|  | 7 | 0 | 13 | 13 |
|  | 8 | 0 | 12 | 12 |
|  | 9 | 3 | 0 | 3 |
|  | 10 | 0 | 2 | 2 |
|  | 11 | 13 | 0 | 13 |
|  | 12 | 0 | 13 | 13 |
|  | 13 | 4 | 0 | 4 |
|  | 14 | 0 | 4 | 4 |
|  | 15 | 0 | 9 | 9 |
| Total | | 72 | 68 | 140 |
