# Supplementary material for: Propofol-based intravenous anesthesia is associated with better survival than desflurane anesthesia in pancreatic cancer surgery
Source: PLoS One. 2020 May 21;15(5):e0233598. doi: 10.1371/journal.pone.0233598 (PMC7241788; doi:10.1371/journal.pone.0233598)
Supplement: S3 Table — (DOCX) [file pone.0233598.s003.docx]

1. **Anesthesiologists (n = 15)**

| **Variables in the Equation** | | | | | | | | |
| --- | --- | --- | --- | --- | --- | --- | --- | --- |
|  | B | SE | Wald | df | Sig. | Exp(B) | 95.0% CI for Exp(B) | |
|  |  |  |  |  |  |  | Lower | Upper |
| Anesthesiologists |  |  | 10.435 | 14 | .730 |  |  |  |
| Anesthesiologists(1) | .698 | .557 | 1.571 | 1 | .210 | 2.010 | .675 | 5.990 |
| Anesthesiologists(2) | .423 | .355 | 1.420 | 1 | .233 | 1.526 | .761 | 3.058 |
| Anesthesiologists(3) | .429 | .477 | .809 | 1 | .368 | 1.535 | .603 | 3.907 |
| Anesthesiologists(4) | -.180 | .748 | .058 | 1 | .810 | .836 | .193 | 3.621 |
| Anesthesiologists(5) | -10.830 | 222.673 | .002 | 1 | .961 | .000 | .000 | 6.850E+184 |
| Anesthesiologists(6) | .586 | .389 | 2.271 | 1 | .132 | 1.796 | .839 | 3.849 |
| Anesthesiologists(7) | .504 | .399 | 1.589 | 1 | .207 | 1.655 | .756 | 3.620 |
| Anesthesiologists(8) | -.062 | .749 | .007 | 1 | .934 | .940 | .217 | 4.076 |
| Anesthesiologists(9) | 1.160 | .754 | 2.367 | 1 | .124 | 3.190 | .728 | 13.982 |
| Anesthesiologists(10) | -.491 | .476 | 1.066 | 1 | .302 | .612 | .241 | 1.555 |
| Anesthesiologists(11) | .358 | .400 | .800 | 1 | .371 | 1.430 | .653 | 3.130 |
| Anesthesiologists(12) | .112 | .627 | .032 | 1 | .859 | 1.118 | .327 | 3.822 |
| Anesthesiologists(13) | .631 | .557 | 1.285 | 1 | .257 | 1.879 | .631 | 5.595 |
| Anesthesiologists(14) | .483 | .450 | 1.154 | 1 | .283 | 1.621 | .671 | 3.915 |
