# Supplementary material for: Propofol-based intravenous anesthesia is associated with better survival than desflurane anesthesia in pancreatic cancer surgery
Source: PLoS One. 2020 May 21;15(5):e0233598. doi: 10.1371/journal.pone.0233598 (PMC7241788; doi:10.1371/journal.pone.0233598)
Supplement: Attachment — Submitted filename: Reply_template_PONE_R1.doc [file pone.0233598.s004.doc]

**Reply to the reviewers’ comments**

| Reviewer Number | Original comments of the reviewer | Reply by the author(s) | Changes done on page number and line number |
| --- | --- | --- | --- |
| 1 | 1. Introduction: You report that “Until now, very few studies have compared the effects of the use of desflurane versus propofol anesthesia on patient outcomes after pancreatic cancer surgery”. Please reference the respective work and better explain in the discussion what your study adds to the literature. | Thanks for your suggestion, we have referenced the respective work and explained in the discussion section. | P18, Line 1-5. |
| 1 | 2. You performed a retrospective analysis. Therefore, the nature of your study is exploratory and the findings are descriptive and hypothesis generating. Throughout the entire manuscript please avoid any wording suggesting a causal relationship between type of anesthesia and outcome. For example, Abstract: Use: “propofol anesthesia was associated with improved survival” instead of “propofol anesthesia improved survival”. | Thanks for your kindly reminder, we revised the manuscript as you suggested. |  |
| 1 | 3. The authors excluded patients who received sevoflurane anesthesia. However, it would be of great interest whether the observed effects are specific for all volatile anesthetics or if they are restricted to desflurane. Sevoflurane Data should be reported. | In fact, there was no patient receiving sevoflurane anesthesia before and after exclusion.  Therefore, no sevoflurane data could be reported. | Figure 1. |
| 1 | 4. Introduction: Reference one is not sufficient to claim that pancreatic cancer has the worst prognosis of any cancer. Also, I am not sure if this is actually the case. How about malignant melanoma or glioblastoma? | Thanks for your kindly reminder, we have revised the sentence as” Pancreatic cancer carries the **poor** prognosis with a median survival of 6 months, and a 5-year survival rate is only around 5%.” | P9, Line 6. |
| 1 | 5. Were your 140 patients cases? If so, please add this information on page 11, (Participants and Data Sources) If not, how did you select individuals included in your analysis. | Yes, our 140 patients were consecutive cases, and we add this information on P11. | P11, Line 9. |
| 1 | 6. P 11: “3–4 μg /mL in FiO2 of 100% oxygen at a flow rate of 300 mL/min”. FiO2 of 100% oxygen does not make sense for i.v. infusion of propofol. | Thanks for your kindly reminder, but we did use FiO2 of 100% oxygen in the two groups intraoperatively. (Anesthesiology 2018;129:932-941, Br J Anaesth. 2019;123:151-160, PLoS One 2019;14:e0224728.) |  |
| 1 | 7. I recommend optimizing the consort diagram (figure 1): How many surgeries for pancreatic cancer were conducted in total in the study hospital between 2005 and 2019? How many patients were excluded due to use of other anesthetics (e.g. sevoflurane). This information would help to evaluate the expertise of the surgical center in sight of pancreatic cancer surgery. | Thanks for your suggestion, we have revised the Figure 1 as your advice. | Figure 1. |
| 1 | 8. Since it is known that surgical resection margins have significant influence on pancreatic cancer outcome I recommend to doublecheck the two groups (propofol vs desflurane) for differences in surgical resection margins (R0 vs R1 resection). If available, data about R0 vs R1 resection margins should be added to the study group characteristics in Table 1. | R0/R1 resection is highly correlated with TNM stage, therefore, R0/R1 resection was not included in the model. | Tables 1 and 2. |
| 1 | 9. I recommend to separate baseline study characteristics from study outcome parameters (e.g. postoperative recurrence/metastasis) in Table 1 and show outcome parameters separately. | Thanks for your suggestion, we do take your suggestion into consideration seriously. But for the flow of data presentation, we prefer to keep the clinical outcomes in Table 1.  Also, we rename the Table 1 as “Patients’ and Treatment Characteristics and **Clinical Outcomes** for Overall Group and Matched Group after Propensity Scoring.” | Table 1. |
| 1 | 10. Was there a specific protocol for postsurgical follow up? If a protocol exists please add information on whether CT scans were performed at pre-defined time intervals or if they were prompted on clinical suspicion. | In our hospital, we follow the pancreatic ductal adenocarcinoma (PDAC) guidelines, and routinely combine tumor marker testing and computed tomography (CT) imaging every three to six months during the first two years after pancreatic cancer surgery. (Eur J Surg Oncol 2019;45:1770-1777) |  |
| 1 | 11. If available, please give information on the applied postoperative chemotherapies and whether there were differences in the two groups between the used chemotherapy regimen. | In our hospital, we routinely use Gemzar (gemcitabine) as the postoperative chemotherapy for pancreatic cancer patients. And there was no difference in the two groups between the used chemotherapy regimen. (Am Fam Physician. 2014;89:626-32) |  |
| 1 | 12. What is the surgeon factor? (page 15) | High surgeon volumes are associated with positive patient outcomes in pancreatic cancer surgery, therefore, we conducted further adjustment for the surgeon factor. ( Ann Surg. 2016;263:727-32) | 1. Table 2. 2. P15, Line 7. |
| 1 | 13. Data from table 3 are mentioned in abstract and manuscript but are not explicitly referred to in the text. Please add “(see Table 3 etc)” and make sure every table and diagram is named and referred to in the text. | Thanks for your kindly reminder, we have revised the manuscript as you suggested. |  |
| 1 | 14. Avoid the expression “marginally significant” | Thanks for your kindly reminder, we have revised the manuscript as you suggested. |  |
| 1 | 15. Due to the small sample size of approx. 10 surgeries per year I would like the authors to give more information on comparability of the study cohort with regard to surgical outcome and survival rates. | We conducted calendar period, and derived same findings if “time since the earliest included patient” was replaced (Supplementary Table 1). | Tables 1 and 2, and Supplementary Table 1. |
| 1 | 16. page 15: please avoid the term “insignificant interaction”. Better: “We did not observe an interaction…” | Thanks for your suggestion, we have revised the manuscript as you suggested. | P16, Line 1-4. |
| 1 | 17. discussion: avoid presenting new data in the discussion (“disease progression in the matched propofol group(62,1%). | Thanks for your suggestion, we have revised the manuscript as you suggested. | P17, Line 9-11. |
| 2 | 1. I think diabetes and diabetic medication and other chronic medications (like metformin) might influence the survival. Do you have any data about it? | We reconducted univariable and multivariable analyses, and only preoperative use of metformin affected the survival (diabetes history did not affect the survival). | Tables 1 and 2. |
| 2 | 2. Regarding the Kaplan-Meier curves, the anesthetic techniques influence the long term outcome (after 3 years), could you give the number of the patients in risk in both groups below the diagram? Do you have an explanation for this phenomenon i.e. long term effect of the anesthetics? | 1. As revised Figure 2.  2. Volatile anesthetics impair numerous immune functions. In contrast, propofol used for total intravenous anesthesia may be protective through its anti-inflammatory and antioxidant properties. Besides, it seems biologically implausible that something as complicated as cancer can be reduced by more than a factor-of-2 simply by anesthetic selection. (Anesthesiology 2019;130:3-5, Anesthesiology 2019;131:219-220) | Figure 2. |
| 2 | 3. Have you performed epidurals in the perioperative period? | There was no epidural anesthesia/analgesia in the two groups in the perioperative period. |  |
| 2 | 4. Have you seen any random or fix effect of the different techniques during the years (have you changed the practice or was the choose of anesthetics left for individual discretion?) | We have not changed the practice and the anesthetic technique was decided by the anesthesiologist’s personal preference. Besides, anesthesiologist factor did not affect postoperative mortality (Supplementary Tables 2 and 3). | Supplementary Tables 2 and 3. |
