# Supplementary material for: Propofol-based intravenous anesthesia is associated with better survival than desflurane anesthesia in pancreatic cancer surgery
Source: PLoS One. 2020 May 21;15(5):e0233598. doi: 10.1371/journal.pone.0233598 (PMC7241788; doi:10.1371/journal.pone.0233598)
Supplement: Attachment — Submitted filename: Reply_template_PONE_R2.doc [file pone.0233598.s005.doc]

**Reply to the reviewers’ comments**

| Reviewer Number | Original comments of the reviewer | Reply by the author(s) | Changes done on page number and line number |
| --- | --- | --- | --- |
| 1 | 1. You added numbers at risk to diagram 2A-C as recommended by Reviewer2. Please double check Figure 2 A-C: If I am not mistaken the diagram does not match with the underlaying values. | Thanks for your kind reminder, we have revised the Figure 2B and 2C. | Figure 2. |
| 1 | 2. Please check page 11:"3–4 μg /mL in FiO2 of 100% oxygen at a flow rate of 300 mL/min”. FiO2 of 100% oxygen does not make sense for i.v. infusion of Propofol. The wording is still confusing and we would recommend to separately describe Infusion concentrations of Propofol and inspiratory Oxygen fraction. | Thanks for your kind reminder, we have revised the manuscript as you suggested. | P11, Line 23 - P12, line 1. |
| 1 | 3. Your comment from response to reviewers: "In our hospital, we follow the pancreatic ductal  adenocarcinoma (PDAC) guidelines, and routinely  combine tumor marker testing and computed  tomography (CT) imaging every three to six months  during the first two years after pancreatic cancer  surgery. (Eur J Surg Oncol 2019;45:1770-1777)"  please also add this information in the text (e.g. methods"). Since metastasis occurance/tumor recurrence is monitored via CT/Tumor markers (time of diagnosis = time of diagnostic) it is important to know if all patients followed the same postoperative procedure. Otherwise it can not be distinguished whether metastasis occurance/tumor recurrence really occurs later in the propofol group or if the CT is just conducted later. | Thanks for your suggestion, we have added this information in the manuscript. | P13, Line 4-7. |
| 1 | 4. Your comment from response to reviewers: "In our hospital, we routinely use Gemzar (gemcitabine) as the postoperative chemotherapy for pancreatic cancer patients. And there was no difference in the two groups between the used chemotherapy regimen. (Am Fam Physician.  2014;89:626-32)". Please add this information to the manuscript. | Thanks for your suggestion, we have added this information in the manuscript. | P13, Line 7-9. |
| 1 | 5. Page 16 line 1ff: Please double check "We did not observe an interaction between the anesthestic....(p=0.733) etc.: these values are not shown in table 3 as you refer to! Please add this to table 3 or adjust the text. | Thanks for your kind reminder, we have revised the manuscript and added these values in table 3. | 1. Page 17, Line 1-6. 2. Table 3. |
